# RNAi-mediated silencing of the *Bmi-1* gene causes growth inhibition and enhances doxorubicin-induced apoptosis in MCF-7 cells

**DOI:** 10.1590/S1415-47572009005000092

**Published:** 2009-12-01

**Authors:** Xiang-mei Wu, Xing Liu, You-quan Bu, Joyeeta Sengupta, Hong-juan Cui, Fa-ping Yi, Tao Liu, Chen-fu Yuan, Yan-yan Shi, Fang-zhou Song

**Affiliations:** 1 Department of Biochemistry and Molecular Biology, Chongqing Medical University, ChongqingPR China; 2 Department of Pediatric Urology, Chongqing Children's Hospital, Chongqing Medical University, ChongqingPR China; 3 Department of Biochemistry and Cancer Biology, Medical University of Ohio, OhioUSA

**Keywords:** RNA interference, Bmi-1, retrovirus vector, doxorubicin, breast cancer

## Abstract

The oncogene *Bmi-1* is a member of the Polycomb group gene family. Its expression is found to be greatly increased in a number of malignant tumors including breast cancer. This could suggest Bmi-1 as a potent therapeutic target. In this study, RNAi was introduced to down-regulate the expression of *Bmi-1* in a highly malignant breast adenocarcinoma cell line, MCF-7. A thorough study of the biological behavior and chemosensitivity changes of the MCF-7 cells was carried out in context to the therapeutic potential of Bmi-1. The results obtained indicated that siRNA targeting of *Bmi-1* could lead to an efficient and specific inhibition of endogenous Bmi-1 activity. The mRNA and protein expression of *Bmi-1* were determined by RT-PCR and Western blot, respectively. Furthermore, silencing of *Bmi-1* resulted in a drastic inhibition of the growth of MCF-7 cells as well as G_1_ /S phase transition. The number of target cells was found to increase in phase G _0_ /G _1_ and decrease in the S phase, but no increase in the basal level of apoptosis was noticed. On the other hand, a reduction in the expression of cyclin D1 and an increase in the expression of p21 were also noticed. Silencing of *Bmi-1* made the MCF-7 cells more sensitive to the chemotherapeutic agent doxorubicin and induced a significantly higher percentage of apoptotic cells. Here, we report on a study regarding the RNAi-mediated silencing of the *Bmi-1* gene in breast cancer.

## Introduction

Breast cancer is one of the most common malignancies affecting women worldwide. Despite the recent development of various therapeutic strategies, the prognosis for this cancer still remains poor. Thus, more efforts are needed to understand its molecular pathway in order to develop an effective therapy to achieve cure.

Bmi-1 (B-cell-specific moloney murine leukemia virus insertion site 1) was originally identified as an oncogenic partner of c-Myc in murine lymphomagenesis (Haupt *et al.*, 1993; Van Lohuizen *et al.*, 1991). It is a component of the Polycomb repressive complex 1, which represses gene expression through chromatin modifications (Valk-Lingbeek *et al.*, 2004). Previous studies have revealed that Bmi-1 is involved in the regulation of stem-cell-associated genes to control cell self-renewing and differentiation (Park *et al.*, 2003; Molofsky *et al.*, 2005). Other studies also demonstrated that Bmi-1 regulates the differentiation and clonogenic self-renewal of I-type neuroblastoma cells (Cui *et al.*, 2006).

*Bmi-1* is overexpressed in several malignancies, such as non-small cell lung cancer (Vonlanthen *et al.*, 2001), colorectal cancer (Kim *et al.*, 2004a), nasopharyngeal carcinoma (Song *et al.*, 2006) and oral cancer (Kang *et al.*, 2007). On the other hand, loss of *Bmi-1* with RNA interference (RNAi) was effective in suppressing growth and tumorigenicity of cancer cells (such as SH-SY5Y neuroblastoma and ovary adenocarcinoma) (Liu *et al.*, 2006a). This is consistent with the study of Cui *et al.* (2007) that showed that down-regulation of *Bmi-1* impaired the ability of neuroblastoma cells to grow in soft agar and tumorigenicity in immunodeficient mice. Therefore, silencing of *Bmi-1* may be of great importance in the design and development of anticancer therapy. However, to our best knowledge, there is so far no study in the literature on the therapeutic potential of Bmi-1 in breast cancer cell lines.

Notably, Bmi-1 was found to regulate self-renewal of breast cancer stem cells and to alter mammary development in a humanized nonobese diabetic-severe combined immunodeficient (SCID) mouse model (Liu *et al.*, 2005; 2006b). Overexpression of *Bmi-1* induces telomerase activity and immortalizes human mammary epithelial cells (MECs), suggesting a potential role for Bmi-1 in the development of human breast cancer (Dimri *et al.*, 2002). Amplification of the *Bmi-1* gene is detected in approximately 85% of human invasive ductal breast cancers and this genomic alteration is correlated with axillary lymph node metastases and positive estrogen receptor status (Kim *et al.*, 2004b). Furthermore, the Bmi-1 mRNA transcript levels are higher in plasma from breast carcinoma patients than in healthy controls, and this amplification of Bmi-1 is predictive of poor clinical outcome (Silva *et al.*, 2007).

Doxorubicin has emerged as one of the most widely used chemotherapeutic agents for breast cancer. Although there is initial response to this chemotherapeutic agent, resistance is seen to develop over a period of time. This leads to a phenotypically more aggressive cell variant with an inclination to metastasize. So, increasing chemosensitivity is very important for breast cancer therapy.

A study on *Bmi-1* mRNA levels in several breast cancer cell lines (MCF-7, MDA-MB-468, MDA-MB-231, T47D) was carried out. In MCF-7 and MDA-MB-231 *Bmi-1* expression was found to be maximal. In order to investigate the possibility of turning Bmi-1 into a novel therapeutic agent for the treatment of breast cancer, MCF-7 was chosen to silence the expression of *Bmi-1* with the highly specific post-transcriptional suppression of RNAi. Thereafter, proliferation, cell cycle status, apoptosis, and chemosensitivity to doxorubicin were also studied. The results obtained suggest that targeting of Bmi-1 may be used as a potential and specific therapeutic tool for the treatment of breast cancer.

## Materials and Methods

###  Cell line and reagents

The highly malignant human breast adenocarcinoma cell line MCF-7 was obtained from Dr. Yin (Third Military Medical University, China). The human *Bmi-1* gene short hairpin RNA (shRNA)-expressing plasmid pSuper-retro/Bmi-1 si and a control plasmid pSuper-retro/GFP si expressing Green Fluorescent Protein (GFP) shRNA (Cui *et al.*, 2007) were used. The Bmi-1 siRNA sequence was 5'-AATGGACATACCTAATACT-3', position 546 to 564 bp relative to the start codon. The GFP target sequence was 5'-GCAAGCTGACCCTGAAGTTC-3'. Lipofectamine 2000 Reagent was purchased from Invitrogen (USA), 3-(4,5-Dimethylthiazol-2-yl)-2,5-diphenyltetrazolium bromide (MTT) and doxorubicin hydrochloride were purchased from Sigma (USA).

###  Cell culture and gene-silencing assays

The MCF-7 cell line was maintained as a monolayer culture in RPMI-1640 medium (Hyclone, USA) with L-glutamine (2 mM) and 10% fetal calf serum (Hyclone, USA) in a humidified atmosphere supplemented with 5% CO_2_ at 37 °C. When the density of cells reached 90%-95%, transfections were carried out with Lipofectamine 2000 reagent, using 2 mg of expression vector/mL serum-free medium, as recommended by the manufacturer. The cells which were transfected with pSuper-retro/Bmi-1 si were named Bmi-1si, and the cells transfected with pSuper-retro/GFP si were named GFPsi. The MCF-7 cells devoid of transfection were used as blank control. After 6 h of transfection, the medium was replaced by serum-containing medium.

###  RNA preparation and semiquantitative RT-PCR

After 48 h of transfection, the total RNA of the cells was extracted using the Trizol method (Invitrogen, USA) according to the manufacturer's directions, and quantified by spectrophotometry at 260 nm. cDNA was generated from 3-5 μg of total RNA, using SuperScript II reverse transcriptase and random primers, according to the manufacturer's protocol (Invitrogen, USA). PCR-based amplification with rTaq DNA polymerase (Takara, Japan) was performed under standard conditions. GAPDH was used as internal control of integrity and uniformity of the sample preparation. The PCR conditions maintained were as follows: pre-denaturing at 94 °C for 5 min, followed by 30 reaction cycles at 94 °C for 30 s, 51 °C for 30 s, 72 °C for 30 s, and a final extension cycle at 72 °C for 5 min. The PCR products were analyzed on a 1.5% agarose gel stained with ethidium bromide, and the resultant target bands were densitometrically analyzed by using Vistra Fluor Imager SI (Molecular Dynamics Inc., USA). The ratio of target gene to GAPDH OD value was used to represent the relative intensity of the target product. Primers were designed with Primer Premier 5.0 software, according to the human sequences obtained from Medline. The primers were designed as follows: Bmi-1, 5'-GACCACTACTGAATATA AGG-3' (sense), and 5'-CATTTGTCAGTCCATCTCTC-3' (anti-sense); GAPDH, 5'-CATGAGAAGTATGACAA CAGCCT-3' (sense), and 5'-CGTCCTTCCACGATA CCAAAGT-3' (anti-sense).

###  Immunofluorescent staining

Cells were grown on glass coverslips and transfected with retrovirus vectors. 48 h after transfection, the culture medium was aspirated and the cells were fixed with 4% paraformaldehyde in phosphate-buffered saline (PBS) for 15 min and permeabilized in 0.2% Triton X-100 in PBS for 5 min at room temperature. Cells were blocked with 3% BSA in PBS for 1 h at room temperature and then incubated with Bmi-1 antibody (1:100 dilution, Upstate, USA) at 4 °C overnight. Then, the cells were washed in PBS (310 min) and incubated with a secondary Rhodamine-conjugated antibody (1:100 dilution, Zhongshan, China) at 37 °C for 1 h. The cells were washed in PBS (410 min), followed by addition of the mixture of ProLong mounting medium and ProLong Antifade reagent onto the slides. Fluorescent images were taken by confocal laser-scanning microscopy using a Leica TCS SP confocal system attached to a Leica DM IRBE microscope; 568 nm laser wavelength was used for analysis.

###  Western blot analysis

Total protein was isolated and quantified. Cells transfected for 48 h were harvested, washed with ice-cold PBS and lysed in lysis buffer containing 25 mM Tris-HCl, pH 8.0, 137 mM NaCl, 2.7 mM KCl, 1% Triton X-100 and protease inhibitor mixture (Sigma, USA) at 4 °C for 30 min, followed by brief sonication. All the experimental steps were performed on ice. After centrifugation at 16,220 g at 4 °C for 10 min, supernatants were collected, and the protein concentration was measured with the BCA assay reagent (Bioteke, China) according to the manufacturer's protocol. Following sodium dodecyl sulfate-polyacrylamide gel electrophoresis (SDS-PAGE), the proteins were transferred onto a PVDF membrane (Immobilon, USA). After saturation, the membranes were incubated at room temperature for 2 h in TBS with 0.1%Tween-20 (TBS-T) containing 5% nonfat dry milk, and subsequently incubated with primary antibodies against Bmi-1 (1:600 dilution), cyclin D1 and p21 (1:200 dilution, Santa Cruz, USA) overnight at 4 °C. Peroxidase conjugated IgG antibodies (MultiSciences Biotech, China) were used as secondary antibodies. The protein was detected using the ECL detection kit (keygen, China) following the manufacturer's protocol. β-actin immunoblotting was used as protein loading control.

###  MTT assay

The MTT assay was used to detect the effect of plasmids on the growth of MCF-7 cells and to determine the 50% inhibitory concentration (IC_50_) of doxorubicin. For measurement of cell growth curves, cells (110^4^/well) were plated into 96-well plates and allowed to grow for 4 days after transfection with pSuper-retro/Bmi-1 si and pSuper-retro/GFP si. Growth curves were plotted as optical density (570 nm) versus days after transfection. To study the IC_50_, 48 h after transfection, cells were treated with various concentrations of doxorubicin (0.03-100 μg/mL) for 72 h. At each time point, 10 μL of MTT stock solution (5 mg/mL in PBS) were added to each microtiter well and incubated for 4 h at 37 °C. After aspiration of the medium, 150 μL of dimethyl sulfoxide was added and mixed, and absorbances were measured at a wavelength of 570 nm. The rate of cell growth inhibition (IR) was calculated according to the following equation: IR = [1–A_570_ (drug)/A_570_ (control)] 100%, where A_570_ (drug) is the absorbance of the cells exposed to doxorubicin and A_570_ (control) is the absorbance of the cells without doxorubicin treatment.

###  Cell cycle assay

The cell cycle was analyzed by using flow cytometry (FCM) with propidium iodide staining. Both floating and attached cells were collected by trypsin digestion and low-speed centrifugation, washed with cold PBS, and then fixed in ice-cold 70% ethanol overnight. The fixed cells were collected by brief centrifugation and resuspended in PBS, after which the cells were treated with RNaseA and stained with propidium iodide for 1 h at room temperature, and finally analyzed by FCM.

###  Apoptosis assay

Apoptosis was assayed using the Annexin V-FITC Apoptosis Kit (keygen, China) according to the manufacturer's instructions. Briefly, the cells were harvested and washed twice with PBS, followed by resuspension in Annexin-V binding buffer, and then FITC-conjugated Annexin V and PI were added. After incubation for 10 min at room temperature in the dark, another binding buffer was added, and the samples were immediately analyzed using FCM.

###  Statistical analysis

All experiments were performed at least three times and statistical analysis was done using the SPSS13.0 package (SPSS Inc., Chicago, USA). The values were expressed as mean ± SD. The ANOVA test was used whenever more than two groups were compared, and the significance level was set at p < 0.05. Dunnett's *post hoc* test was used to analyze multiple comparisons. *P* values of less than 0.05 (p < 0.05) were considered to be statistically significant.

## Results

###  Inhibition of Bmi-1 expression at the mRNA and protein levels

**Figure 1:**
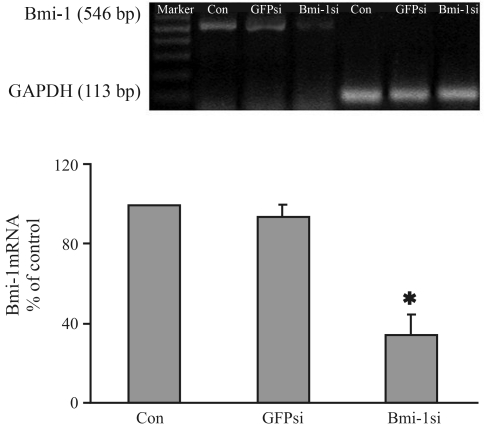
RT-PCR analysis of Bmi-1 mRNA level of MCF-7 cells 48 h after transfection. The ratio of band density for Bmi-1 to GAPDH in Bmi-1si was significantly lower, showing a 67% reduction in density as compared to the blank control; in GFPsi, the ratio showed a 5.2% reduction compared to the blank control. There were significant differences between Bmi-1si and controls (p < 0.05). Bar graphs represent the results of the densitometry analysis of Bmi-1, expressed as a percentage of the ratio (molecule of interest/GAPDH mRNA) detected in the control (*p < 0.05 *vs.* control).

**Figure 2:**
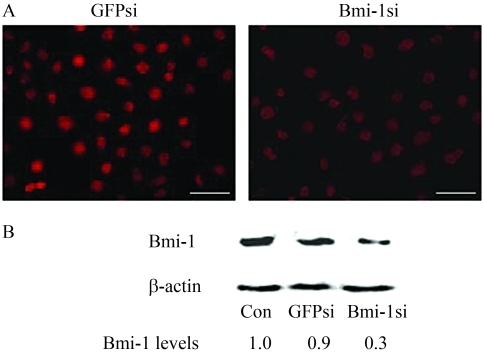
Effect of *Bmi-1* silencing on the expression of Bmi-1 protein in MCF-7 cells. (A) immunofluorescent staining of Bmi-1 48 h after transfection, showing a significantly weaker fluorescence in MCF-7 cells of Bmi-1si compared to the blank control and GFPsi (Scale bars, 100 μm). (B) Western blot analysis of Bmi-1 expression 48 h after transfection, showing a reduction of up to 70% in the protein level of Bmi-1, clearly indicating a marked difference from that of the blank control and GFPsi (p < 0.05).

In the first phase of our study, we determined the silencing of the *Bmi-1* gene activity in the targeted MCF-7 cells. MCF-7 cells were transiently transfected with pSuper-retro/Bmi-1 si and pSuper-retro/GFP si. After transfection for 48 h, total RNA and whole-cell lysates were prepared and subjected to semiquantitative RT-PCR and Western blot, respectively. Figure 1 clearly indicates that the Bmi-1/GAPDH ratio of band density in Bmi-1si was significantly lower and also shows a 67% reduction in density compared to the blank control. In GFPsi, the same ratio showed a 5.2% reduction compared to the blank control. The differences between Bmi-1si and controls were statistically significant (p < 0.05). Similarly, the protein level of Bmi-1 in Bmi-1si showed a reduction of up to 70%, clearly indicating a marked difference from that of the blank control and GFPsi (p < 0.05) (Figure 2B). Compared with the blank control and GFPsi, immunofluorescent staining showed a significantly weaker fluorescence in the Bmi-1si MCF-7 cells (Figure 2A).

### *Bmi-1* silencing inhibited MCF-7 cell proliferation

**Figure 3:**
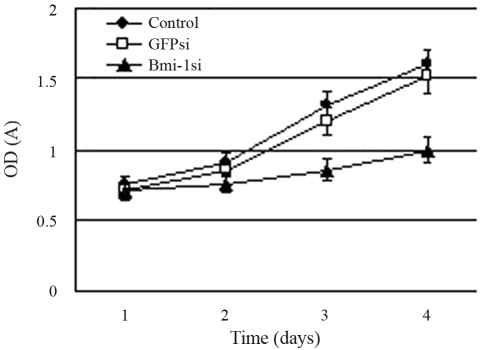
Effect of *Bmi-1* silencing on proliferation of MCF-7 cells. Growth curves of MCF-7 cells were analyzed by MTT assay. At 24 h and 48 h after transfection, the optical density (570 nm) in Bmi-1si showed no significant differences compared to the blank control and GFPsi. However, 72 h after transfection, there were significant differences between Bmi-1si and controls. The MTT assay indicated strong inhibition in the growth rate of MCF-7 cells in Bmi-1si. Each value represents the mean standard deviation (SD) of triplicate cultures.

The MTT assay indicated strong inhibition in the growth rate of Bmi-1si MCF-7 cells compared to that of the blank control and GFPsi. After 24 h and 48 h of transfection, the optical density (570 nm) in Bmi-1si showed no significant differences compared to the blank control and GFPsi. However, 72 h after transfection, there were significant differences between Bmi-1si and controls (Figure 3).

### *Bmi-1* silencing induced G_0_/G_1_cell cycle arrest and abnormal expression of cyclin D1 and p21

**Figure 4:**
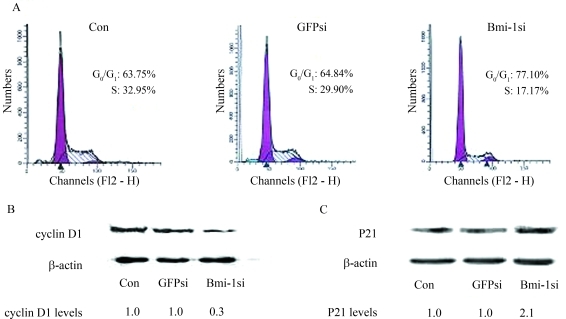
Effect of *Bmi-1* silencing on cell cycle progression and expression of cyclin D1, p21 of MCF-7 cells. (A) The effect of *Bmi-1* silencing on cell cycle progression as shown by FCM. Cells were harvested 48 h after transfection and then stained with propidium iodide (PI). *Bmi-1* silencing induced an obvious increase in the number of cells at G0/G1 phase and reduction in S phase; 77.10% of the MCF-7 cells in Bmi-1si were found at G_0_/G_1_ phase, compared to 63.75% and 64.84% cells in blank control and GFPsi, respectively. There were significant differences between Bmi-1si and controls (p < 0.05). (B) and (C) Western blot analysis of cyclin D1 and p21 in MCF-7 cells 48 h after transfection. Relative levels of protein are indicated. β-actin levels are shown as loading control. Compared with blank control and GFPsi, a reduction in the expression of cyclin D1 and an increase in the expression of p21 were also noticed (p < 0.05).

The effect of *Bmi-1* silencing on cell cycle progression was analyzed using FCM. *Bmi-1* silencing induced an obvious increase in the number of cells at G_0_/G_1_ phase and reduction in S phase, as 77.10% of the MCF-7 cells in Bmi-1si were noticed at G_0_/G_1_ phase, compared to 63.75% and 64.84% cells in the blank control and GFPsi, respectively (Figure 4A). There were significant differences between Bmi-1si and controls (p < 0.05). Western blot results clearly showed a reduction in the expression of cyclin D1 and an increase of p21 in Bmi-1si compared to the blank control and GFPsi (Figure 4B, C).

###  Apoptosis not induced by *Bmi-1* silencing

**Figure 5:**
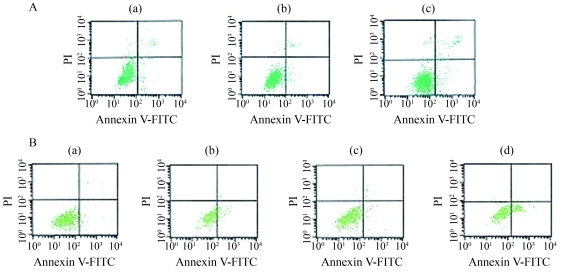
(A) Effect of *Bmi-1* silencing on cell apoptosis in MCF-7 cells. Cells were harvested 48 h after transfection and then stained with Annexin/PI for apoptosis detection. (a) Blank control, (b) GFPsi, (c) Bmi-1si. The basal level of apoptosis in Bmi-1si was 4.94%, while in the blank control and GFPsi it was 3.18% and 2.84, respectively. No significant differences in cell apoptosis were noticed among these three groups. (B) Effect of *Bmi-1* silencing on doxorubicin-induced apoptosis. The apoptotic rate was examined in cells after treatment with 1 μg/mL doxorubicin for 48 h. (a) Blank control, (b) blank control treated with 1 μg/mL doxorubicin, (c) Bmi-1si, (d) Bmi-1si treated with 1 μg/mL doxorubicin. The level of apoptosis in the blank control and Bmi-1si was 6.70% and 6.72%, respectively. The apoptotic rate in Bmi-1si treated with doxorubicin was 37%, while in the blank control treated with doxorubicin it was 21%. This difference was significant (p < 0.05).

The apoptotic rate was examined in three groups 48 h after transfection and the results are shown in Figure 5A. The basal level of apoptosis in Bmi-1si was 4.94%, compared to 3.18% and 2.84% in the blank control and GFPsi, respectively. No significant differences in cell apoptosis were noticed among these three groups.

### *Bmi-1* silencing increased doxorubicin-induced apoptosis

The apoptotic rate was further examined in cells after treatment with 1 μg/mL doxorubicin for 48 h. The FCM data showed that the apoptotic rate in Bmi-1si treated with doxorubicin was 37%, as compared to 21% in the blank control also with doxorubicin. The levels of apoptosis in the blank control and Bmi-1si were 6.70% and 6.72%, respectively (Figure 5B). The differences between Bmi-1si treated with doxorubicin and the blank control treated with doxorubicin were significant (p < 0.05).

### *Bmi-1* silencing made the cells more sensitive to doxorubicin

**Figure 6:**
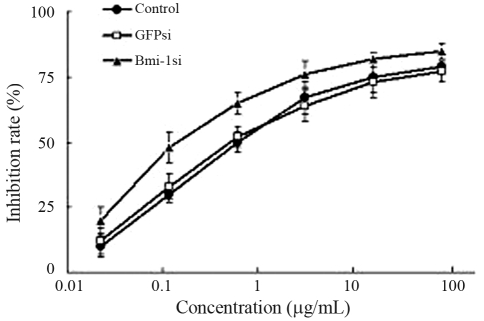
Effect of *Bmi-1* silencing on the chemosensitivity of MCF-7 cells to doxorubicin. The Y-axis indicates IR, the X-axis indicates concentration of doxorubicin. Cells in Bmi-1si showed higher IR than that in the blank control and GFPsi. The IC_50_ value of doxorubicin in Bmi-1si was 0.12 ± 0.07 μg/mL, compared to 0.86 ± 0.02 μg/mL and 0.84 ± 0.02 μg/mL in the blank control and GFPsi, respectively. The differences between the IC_50_ of Bmi-1si and that of controls were significant (p < 0.05).

After treatment with various concentrations of doxorubicin for 72 h, we found that the Bmi-1si cells showed a higher IR than the blank control and GFPsi. The IC_50_ value of doxorubicin in Bmi-1si was 0.12 ± 0.07 μg/mL, compared to 0.86 ± 0.02 μg/mL in the blank control and 0.84 ± 0.02 μg/mL in GFPsi, respectively (Figure 6). There were significant differences between the IC_50_ of Bmi-1si and that of controls.

## Discussion

Previously, BE(2)-C cells infected with Bmi-1 siRNA expressing retrovirus showed a 70%-80% reduction in the Bmi-1 protein level (Cui *et al.*, 2006). In the present study, the same retrovirus vector was used to transiently transfect MCF-7 cells, resulting in 67% and 70% reductions of Bmi-1 mRNA and protein levels, respectively. These data indicate that the RNAi strategy to silence Bmi-1 is specific and effective.

In this study, we found that the growth of MCF-7 cell was significantly retarded by *Bmi-1* silencing. Because the Bmi-1-specific growth retardation was mediated by a delayed cell cycle progression and/or an increased level of apoptosis (Meng *et al.*, 2005; Liu *et al.*, 2006a; Yu *et al.*, 2007), we measured the cell cycle distribution and apoptosis ratio by FCM, in order to determine the relation between inhibition of cell growth and cell cycle arrest or apoptosis.

According to our results, *Bmi-1* silencing both disrupted cell cycle progression and inhibited G_1_-S phase transition significantly. The increase in the number of cells in G_0_/G_1_ phase and decrease in S phase are consistent with the previous results observed in K562 (Meng *et al.*, 2005) and leukemic cells (Lessard and Sauvageau, 2003). Bmi-1 negatively regulates p16^INK4a^ (Jacobs *et al.*, 1999), which acts in the P16^INK4a^-pRb pathway. P16^INK4a^ affects pRb by inhibiting the cyclin D/cyclin-dependent kinase 4/6 kinase complex. In the absence of Bmi-1, p16^INK4a^ may get up-regulated and prevent binding of cyclin-dependent kinase 4/6 to cyclin D, thus inhibiting kinase activity. This in turn results in hypophosphorylation of pRb, which ultimately leads to cell cycle arrest, senescence, or apoptosis, depending on context (Sherr, 2001). Our study shows that the marked down-regulation of cyclin D1 might be related to the inhibition of the G_1_-S phase transition.

Our results indicate that the expression of p21 protein was increased. Bmi-1 can negatively regulate ARF, which probably promotes senescence by regulating the ARF-p53-p21 pathway (Jacobs *et al.*, 1999; Dimri *et al.*, 2000). Evidence has shown the key role of p21 in senescence. Abrogation of the p53-p21 pathway by various strategies can bypass senescence in human and mouse cells, a variety of stimuli induce senescence in a p53/p21-dependent manner and enforced expression of p21 in certain cell types can induce a senescence-like phenotype (Dimri, 2005). So, we conjectured that p21 might be involved in the senescence induced by Bmi-1 silencing.

Bmi-1 may negatively regulate p19ARF, which sequesters the p53 inhibitor MDM2 and thereby prevents the degradation of p53, resulting in p53-mediated apoptosis (Jacobs *et al.*, 1999). So, loss of Bmi-1 may promote cell apoptosis (Liu *et al.*, 2006a). However, in agreement with previous findings in human I-type neuroblastoma cells (Cui *et al.*, 2006) and in A549 lung cancer cells (Yu *et al.*, 2007), we observed no significant cell apoptosis. This suggests that *Bmi-1* silencing affects cell apoptosis, which may vary in different cell lines. We therefore conclude that *Bmi-1* silencing may induce the inhibition of MCF-7 cell proliferation through a mechanism that is largely dependent on cell cycle regulation, but not on apoptosis.

We also studied the role of Bmi-1 on chemotherapy-induced apoptosis in MCF-7 cells and found that the down-regulation of *Bmi-1* resulted in an increased sensitivity of these cells to doxorubicin, expressed by apoptosis. This report is consistent with a previous result observation in nasopharyngeal carcinoma cells (Qin *et al.*, 2008).

Altogether, our study demonstrates that siRNA targeting of *Bmi-1* led to the efficient and specific inhibition of endogenous Bmi-1 mRNA and protein expression of MCF-7 cells *in vitro*. Down-regulation of Bmi-1 inhibited the proliferation and increased the chemosensitivity of MCF-7 cells, indicating that Bmi-1 can be developed into a therapeutic option for the treatment of breast cancer. Further studies will be directed toward uncovering the mechanism underlying the enhancement of doxorubicin-induced apoptosis by the silencing of *Bmi-1*.
